# A longitudinal analysis of the association between the living arrangements and psychological well-being of older Chinese adults: the role of income sources

**DOI:** 10.1186/s12877-019-1371-0

**Published:** 2019-12-10

**Authors:** Zi Zhou, Lun Cai, Meilan Zhuang, Y. Alicia Hong, Ya Fang

**Affiliations:** 1 State Key Laboratory of Molecular Vaccinology and Molecular Diagnostics, School of Public Health, Xiamen University, Xiang’an South Road, Xiamen, Fujian China; 2 Key Laboratory of Health Technology Assessment of Fujian Province University, School of Public Health, Xiamen University, Xiamen, Fujian China; 3 College of Health & Human Services, George Mason University, Fairfax, VA USA

**Keywords:** Living arrangements, Psychological well-being, Income sources, Moderating effect, Older adults

## Abstract

**Background:**

Understanding how living arrangements may affect psychological well-being (PWB) is critical in China, a society with the largest older population in the world. However, few studies have examined the moderating effect of income sources on the relationship between living arrangements and PWB. Our aim was to examine whether living arrangements are associated with PWB and whether income sources moderate this association.

**Methods:**

The data were drawn from the third (2002) to sixth (2011/2012) waves of the Chinese Longitudinal Healthy Longevity Survey (CLHLS). Six questions reflecting older adults’ well-being were used to measure PWB. Living arrangements were classified as follows: living alone, living with family and living in an institution. Income sources were categorized into financially independent, supported by children, and governmental support. We performed random-effects ordinal probit models to examine the association of living arrangements with PWB and the moderating effect of income sources on this relationship.

**Results:**

We included a total sample of 30,899 observations for 16,020 respondents aged 65 and over during 9-year follow-up. Older adults living with family (*β* = .29, *p* < .001) and those living in an institution (*β* = .34, *p* < .001) had stronger PWB than those living alone; moreover, support from children (*β*= −.24, *p* < .001) or from the government (*β*= −.08, *p* < .05) has a negative effect on PWB compared to the effect of financial self-support. Living in an institution with support from children (*β*= −.22, *p* < .05) led to lower PWB than living alone with financial self-support. The opposite result was observed for older adults living with their family and supported by the government (*β* = .16, *p* < .05).

**Conclusions:**

Our analysis provides a significant contribution to the existing literature on the relationship between living arrangements and PWB in China. We recognize that living with family or in an institution leads to better PWB than does living alone. In addition, financial support from the government can moderate this association.

## Background

Psychological well-being (PWB), an important indicator of successful ageing, was conceptualized for assessing mental health across the dimensions of overall quality of life, positivity, perceived happiness, and lack of loneliness among older adults [1]. Studies have shown that improved PWB could be associated with improved quality of life [2], reduced risk of incident chronic diseases [3–5], and prolonged lifespan [6, 7]. An increasing number of studies document an association of living arrangements with PWB among older adults [8–10] . Such studies have mostly been from developed countries, with limited data from developing countries such as China, the country with the largest elderly population [11, 12]. The proportion of older Chinese adults aged 65 and over increased from 7% in 2000 to 10.8% in 2016 and is expected to reach 15.7% by 2030, according to the United Nations [13]. In China, older adults traditionally live with their adult children, typically their sons [14]. Family relationships were continually guided by filial piety, which emphasized physical care, emotional support, respect, and obedience to older adults. There are two hypotheses to explain the effect of living with family. One hypothesis is that older adults who live with family can more easily receive material support, such as assistance in daily life and financial support, and thus experience less isolation and loneliness. The competing hypothesis is that the potential irritations of family life may reduce any advantages of living with family [12].

However, the growing size of the older population, the one-child family planning policy, increased urbanization and massive rural-to-urban migration have significantly changed the traditional family structure [15, 16]. An increasing number of older adults are living by themselves or in senior care facilities, resulting in lower prevalence of traditional family care. Different findings from existing research on the associations between the PWB of older adults and living alone or in an institution show inconsistent evidence. Some studies indicated that those living alone are more depressed and less satisfied with life [17, 18] and more likely to develop disabilities [19]. However, other studies suggested the opposite [20]; elderly people who live alone are reportedly healthier (activities of daily living (ADL), cognitive functions) than those who live with family [21, 22]. In addition, research from Korea, Japan, the United States, and Canada showed that living in an institution, representing a loss of independence, was associated with less happiness and lower quality of life [23]. In China, given the long history of cultural norms and social stigma, institutionalized older adults face adjustment challenges after leaving their community [15]; however, institutions provide care and medical facilities, offering environments that are rich in social interaction and psychological comforts [24].

The relationship between living arrangements and PWB could be moderated by income sources. A recent study from Korea showed that older adults’ PWB was strongly associated with whether or not they received support [25] . Unlike most developed countries with an established pension and welfare system, China introduced its retirement system only three decades ago, and most older Chinese adults, including those living in rural areas, have to rely on either self-support or intergenerational support from children and children-in-law [26]. Furthermore, support from government through a minimum living standard guarantee programme or basic pension insurance has become the most important source of income for poor older adults in China [27, 28]. Different income sources may have different influences on people’s decision making and state of mind and subsequently affect their PWB. For example, older adults with financial self-support have autonomy and independence, which are highly valued in Western culture [29]. Those older adults are more self-reliant and able to make their own decisions while living with adult children. According to the cultural traditions of filial piety, older adults who are supported by children are likely to feel proud and grateful [30], but intergenerational financial support may increase the probability of withdrawal of informal support from their family, in turn decreasing the PWB that can result from living with family [31]. However, few studies have examined the moderating effect of income sources on the relationship between living arrangements and PWB.

Our aim was to examine whether living arrangements are associated with PWB and whether income sources moderate this association. We used a large representative survey of older Chinese adults to address this research question.

## Methods

### Data and sample

The data were drawn from the Chinese Longitudinal Healthy Longevity Survey (CLHLS), which began in 1998. The participant sample was randomly selected from almost 50% of the cities and counties of the 23 provinces in China. Follow-up investigations were conducted in 2000, 2002, 2005, 2008/2009 and 2011/2012, and the later research expanded to individuals who were at least 65 years old since 2002. This survey accumulated comprehensive information on older adults in China, including demographic characteristics, socioeconomic and social support, income sources, health behaviours, health status, and living arrangements via face-to-face interviews. Questions such as PWB and the Mini-Mental State Examination (MMSE) tests were answered by the interviewees only. For the objective and factual questions, the interviewees were required to answer to the best of their ability. If interviewees were not able to answer these questions, a proxy such as a spouse or children provide answers. More information about the CLHLS, including the data quality assessment and sample design method, can be found elsewhere [32].

We used data from the third (2002) to sixth (2011/2012) waves. We restricted the analytic sample to initial observations (2002), thus mitigating the issue of selection bias. The third (2002) wave of the CLHLS included 16,064 respondents. We excluded 44 participants under 65 years of age. A follow-up survey (Wave 4) was conducted in 2005, when almost half of the third-wave respondents (*n* = 8175) were interviewed again. Approximately 36.7% (*n* = 5874) had died, and approximately 12.6% (*n* = 2015) were lost to follow-up. The fifth survey was conducted in 2008–2009, in which 4191 old people survived and were interviewed again. There were only 2513 older adults who survived and were interviewed again in the 2011/2012 wave survey. We excluded those respondents who were deceased or lost to follow-up, resulting in a total sample of 30,899 observations for 16,020 respondents aged 65 and over.

### Variables and measures

#### Dependent variable

The CLHLS included a series of questions on older adults’ quality of life. We used six questions to generate two indices representing older adults’ PWB: one for positive PWB and the other for negative PWB. The items for positive PWB were “How do you think of your life at present?”, “Do you always look on the bright side of things?” and “Are you as happy now as when you were younger?” Five response options (very good, good, so-so, bad and very bad) were given for the three items. Similarly, the three items for negative PWB were “Do you often feel fearful or anxious?”, “Do you often feel lonely and isolated?” and “Do you feel the older you get, the more useless you are?” Five response options (always, often, sometimes, seldom, and never) were given. The scores ranged from 1 (very good or always) to 5 (very bad or never), and we reversed the order of the negative PWB question responses and calculated scores by summing all 6 items so that a higher score for PWB indicated better well-being. The PWB score ranged from 6 to 30, and Cronbach’s alpha for the PWB scale was α = .752, which implied internal consistency. The fit indices of the confirmatory factor analysis model indicated an acceptable fit [33] (root mean square error of approximation (RMSEA) = .075; comparative fit index (CFI) = .965).

#### Independent variables

The independent variables included living arrangements and income sources. Living arrangements were classified as follows: living alone, living with family and living in an institution. Income sources were measured by the question “what’s your main financial source?” The answer included nine options: financially independent (from pension, working for oneself, or spouse), supported by children (from adult children, grandchildren or other relatives), and governmental support (from the local government or community) [31]. The participants were allowed to select one response from the 9 options.

#### Covariates

There were three sets of potential confounders, including socio-demographic characteristics, health behaviours, and health status. Socio-demographic variables included age (in years), residence (rural vs. urban), gender (female vs. male), ethnicity (minority vs. Han), marital status (married vs. unmarried), children alive and sibling alive (yes vs. no), educational level (in years), occupational status (professional occupation vs. others), income (log-transformed because of the skew of the distribution) and financial sufficiency (yes vs. no). Social support was assessed by asking the respondents if they had someone to talk to or to get help from when necessary. Health behaviours included current behaviours of smoking, drinking, and engaging in any physical exercise (yes vs. no).

Health status was measured by three indices: chronic condition, ADL disability, and cognitive functioning. Having a chronic condition was measured by the question “Do you suffer from the following diseases?” The respondents could choose from 22 options, such as hypertension, diabetes, and stroke. Chronic diseases were classified as having no chronic disease, one chronic disease, and two or more chronic diseases. ADL was measured with the Katz Index by six items: bathing, dressing, toileting, indoor transferring, eating, and continence. Disability in ADL was categorized as no ADL limitation, one ADL limitation, and two or more ADL limitations. Cognitive functioning was measured by the Chinese version of the MMSE [34]. Based on prior literature on the CLHLS, older adults with scores less than 18 were considered cognitively impaired [35].

### Data analyses

First, we describe the baseline characteristics of each variable for each kind of living arrangement. The Pearson χ^2^ test or analysis of variance was used to test for significant differences among living arrangements. Second, considering that the outcome variable of PWB was ordinal with a response range from 6 to 30 and considering the longitudinal design of the CLHLS, we performed random-effects ordinal probit models to examine the association of living arrangements with PWB and the moderating effect of income sources on this relationship. A random intercept for each person across time was used to control for the unobserved individual heterogeneity or intra-person variability. Three models were developed. In the first model, we regressed PWB on living arrangements, with adjustment for socio-demographic characteristics, health behaviours, and health status. In the second model, income sources were added to examine whether the additional variables had an effect on PWB. The third model was a full model to examine the moderating effect of income sources on the association between living arrangements and PWB. All analyses were performed using Stata version 13.0 (StataCorp; College Station, TX, USA).

We conducted sensitivity analyses to test the robustness of the random-effects ordinal probit models to sample attrition and proxy response. First, we reanalysed the models with adjustment for a dummy variable to indicate the deceased and follow-up identities. Second, we limited the respondents to those who answered the survey question without any help from others.

## Results

Table 1 lists the characteristics of the baseline sample by living arrangements. The mean age of the respondents was 86.39 years old. The majority (53.97%) of the respondents lived in rural areas, 57.39% were female, 5.59% were minority, and 10.48% had a professional occupation. A total of 80.59% of the older adults were financially sufficient, and 79.30% of those living in an institution were financially self-sufficient. The proportions of the respondents living with family, alone, and in an institution were 81.91, 13.48 and 4.61%, respectively. Most respondents were supported by their adult children (65.22%), while 28.03% were financially independent. Specifically, the main resource of those living alone and living with family was support from their adult children, and only 23.30% of those living alone were financially independent. Moreover, 48.04% of those living in an institution were supported by the government, and fewer than 1/3 were supported by their children. The institutionalized older adults had poorer physical health than those living alone. The average PWB score was 22.80, and the participants living alone had the lowest average scores.

**Table 1:** Baseline characteristics of participants stratified by living arrangements in the CLHLS

Variables	Total	Alone	With family	Institution	*P* Value
	(*n* = 16,020)	(*n* = 2159)	(*n* = 13,122)	(*n* = 739)	
	n (%)	n (%)	n (%)	n (%)	
Socio-demographic factors					
Age, mean (SD)	86.39 (11.66)	86.43 (10.79)	86.27 (11.91)	88.42 (9.08)	< 0.01
Rural	8646 (53.97)	1259 (58.31)	7166 (54.61)	221 (29.91)	< 0.01
Female	9194 (57.39)	1346 (62.34)	7444 (56.73)	404 (54.67)	< 0.01
Minority	895 (5.59)	94 (4.35)	778 (5.93)	23 (3.11)	< 0.01
Married	5005 (31.24)	84 (3.89)	4865 (37.08)	56 (7.58)	< 0.01
Child alive	14,115 (88.11)	1816 (84.11)	11,908 (90.75)	391 (52.91)	< 0.01
Sibling alive	7230 (45.13)	950 (44.00)	6046 (46.08)	234 (31.66)	< 0.01
Education, mean (SD)	2.01 (3.48)	1.69 (3.05)	2.06 (3.52)	2.24 (3.85)	< 0.01
Professional occupation	1679 (10.48)	236 (10.93)	1269 (9.67)	174 (23.55)	< 0.01
Financially sufficient	12,910 (80.59)	1564 (72.0)	10,760 (82.00)	586 (79.30)	< 0.01
Social support	15,688 (97.93)	1951 (90.37)	13,018 (99.21)	719 (97.29)	< 0.01
Health behaviours					
Current smoker	2965 (18.51)	382 (17.69)	2466 (18.79)	117 (15.83)	0.08
Current drinker	3291 (20.54)	403 (18.67)	2757 (21.01)	131 (17.73)	0.01
Regular exercise	5075 (31.68)	601 (27.84)	4167 (31.76)	307 (41.54)	< 0.01
Chronic diseases					< 0.01
No	6137 (38.31)	844 (39.09)	5059 (38.55)	234 (31.66)	
One	5104 (31.86)	640 (29.64)	4234 (32.27)	230 (31.12)	
Two or more	4779 (29.83)	675 (31.26)	3829 (29.18)	275 (37.21)	
ADL disability					< 0.01
No	11,112 (69.36)	1709 (79.16)	8919 (67.97)	484 (65.49)	
One	2101 (13.11)	229 (10.61)	1770 (13.49)	102 (13.80)	
Two or more	2807 (17.52)	221 (10.24)	2433 (18.54)	153 (20.70)	
Cognitively impaired	4581 (28.60)	576 (26.68)	3751 (28.59)	254 (34.37)	< 0.01
Income sources					< 0.01
financial self-support	4490 (28.03)	503 (23.30)	3829 (29.18)	158 (21.38)	
Children support	10,448 (65.22)	1394 (64.57)	8828 (67.28)	226 (30.58)	
Government support	1082 (6.75)	262 (12.14)	465 (3.54)	355 (48.04)	
PWB, mean (SD)	22.80 (4.26)	21.18 (4.46)	23.05 (4.17)	23.13 (4.22)	< 0.01

*Notes. SD* standard deviation, *ADL* activities of daily living, *PWB* psychological well-being

**Fig. 1:**
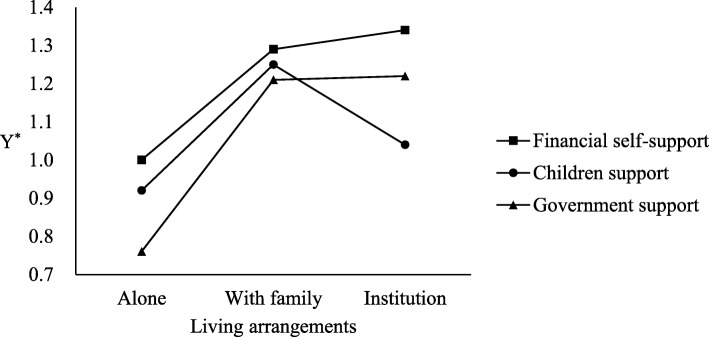
The interaction between living arrangements and psychological well-being

The analysis results of the coefficients estimated from the random-effects ordinal probit model for PWB are presented in Table 2. The significant evidence from likelihood ratio (LR) tests (*P* < .001) indicates that the fit of the models can significantly improve the estimation and control for individual heterogeneity. Model 1 indicates a significantly positive association between PWB and living with family or in an institution compared to living alone. Older adults supported by children or the government scored significantly lower than those who were financially independent (model 2). Model 3 indicates that older adults living with family (*β* = .29, *p* < .001) and those living in an institution (*β* = .34, *p* < .001) had stronger PWB than those living alone; moreover, support from children (*β*= −.08, *p* < .05) or from the government (*β*= −.24, *p* < .001) has a negative effect on PWB compared to the effect of financial self-support. Moreover, the LR test for interaction terms showed that the interacting effects of income sources play a significant role in the relationship between living arrangements and PWB (LR *χ*^*2*^(4) = 14.41, *P* < 0.01). Living in an institution with support from children (*β*= −.22, *p* < .05) led to lower PWB than living alone with financial self-support, and living with family with support from children also led to lower PWB, but this effect was nonsignificant. The opposite result was observed for older adults living with their family and supported by the government (*β* = .16, *p* < .05). Figure 1 shows the interaction between living arrangements and PWB. The results of a stratified analysis by income sources also confirmed the interacting effects of income sources [see Additional file 1: Table S1].

**Table 2:** Coefficients from random-effects ordinal probit models for psychological well-being in the CLHLS

Variables	Psychological well-being		
	Model 1	Model 2	Model 3
Socio-demographic factors			
Age	0.01 (0.01–0.01) ^***^	0.01 (0.01–0.01) ^***^	0.01 (0.01–0.01) ^***^
Wave	−0.00 (− 0.01 - − 0.00) ^*^	−0.00 (− 0.01–0.00)	-0.00 (− 0.01 - -0.00) ^*^
Rural (vs. urban)	−0.15 (− 0.17 - -0.12)^***^	− 0.13 (− 0.15 - -0.10) ^***^	−0.13 (− 0.15 - − 0.10)^***^
Female (vs. male)	-0.10 (− 0.13 - -0.07)^***^	−0.08 (− 0.11 - -0.05) ^***^	−0.08 (− 0.11 - -0.05)^***^
Minority (vs. Han)	0.01 (− 0.05–0.06)	0.01 (− 0.05–0.07)	0.01 (−0.05–0.07)
Married (vs. unmarried)	0.14 (0.11–0.18) ^***^	0.12 (0.08–0.15) ^***^	0.12 (0.08–0.15) ^***^
Child alive (vs. no child alive)	0.08 (0.03–0.13) ^**^	0.07 (0.02–0.12) ^**^	0.08 (0.03–0.12) ^**^
Sibling alive (vs. no sibling alive)	0.05 (0.02–0.08) ^***^	0.05 (0.02–0.08) ^**^	0.05 (0.02–0.08) ^**^
Education	0.00 (0.00–0.01) ^***^	0.00 (0.00–0.00) ^***^	0.00 (0.00–0.00) ^***^
Professional occupation (vs. non-professional)	0.15 (0.10–0.19) ^***^	0.13 (0.09–0.18) ^***^	0.13 (0.09–0.18) ^***^
Financially sufficient (vs. insufficient)	0.50 (0.47–0.53) ^***^	0.49 (0.46–0.52) ^***^	0.49 (0.46–0.52) ^***^
Social support (vs.no)	0.12 (0.06–0.17) ^***^	0.12 (0.07–0.18) ^***^	0.12 (0.06–0.17) ^***^
Health behaviours			
Current smoker (vs. no)	0.04 (0.00–0.07) ^*^	0.04 (0.01–0.08) ^*^	0.04 (0.01–0.08) ^*^
Current drinker (vs. no)	0.10 (0.07–0.13) ^***^	0.10 (0.06–0.13) ^***^	0.10 (0.06–0.13) ^***^
Regular exercise (vs. no)	0.30 (0.27–0.32) ^***^	0.29 (0.26–0.32) ^***^	0.29 (0.26–0.32) ^***^
Health status			
Chronic diseases	−0.08 (− 0.10 - -0.07)^***^	−0.08 (− 0.10 - -0.07) ^***^	−0.08 (− 0.10 - -0.07)^***^
ADL disability	0.02 (− 0.00–0.04)	0.02 (− 0.00–0.04)	0.02 (−0.00–0.04)
Cognitively impaired (vs. non-impaired)	0.50 (0.47–0.54) ^***^	0.51 (0.47–0.54) ^***^	0.51 (0.47–0.54) ^***^
Living arrangements (vs. living alone)			
With family	0.31 (0.27–0.35)^***^	0.31 (0.27–0.35)^***^	0.29 (0.24–0.33)^***^
Institution	0.26 (0.19–0.34)^***^	0.28 (0.20–0.36)^***^	0.34 (0.19–0.49)^***^
Income sources (vs. financial self-support)			
Children support		−0.12 (− 0.15 - -0.09)^***^	−0.08 (− 0.16 - -0.00)^*^
Government support		−0.14 (− 0.20 - -0.08)^***^	−0.24 (− 0.35 - -0.12)^***^
Living arrangements^*^Income sources			
With family^*^children support			0.04 (−0.04–0.13)
With family^*^government support			0.16 (0.03–0.28)^*^
Institution^*^ children support			−0.22 (− 0.41 - -0.02)^*^
Institution^*^ government support			0.12 (−0.08–0.32)
Variance of random effect	0.18 (0.16–0.20)^***^	0.17 (0.15–0.20)^***^	0.18 (0.15–0.20)^***^
LR test	358.36^***^	346.62^***^	346.09^***^

*Notes. ADL* activities of daily living, *LR* likelihood ratio

^*^P < .05, ^**^P < .01, ^***^P < .001

Regarding the effect of the control variables, better PWB was observed for older adults who were older, urban, male, married, and educated; those who had a professional occupation; those who were financially self-sufficient; and those who had social support. Currently, smoking, drinking, regularly exercising, and living with children and siblings were significantly associated with better PWB, while having diseases led to negative feelings. In addition, cognitively impaired adults felt significantly better than normal, which might be the result of their caregivers helping them answer the questions. The coefficients of the variables were mostly robust among the three models [Table 2 near here]. The results of sensitivity analyses indicated that there was no significant change after adjustment for the dummy variable indicating an individual was deceased or lost to follow-up or after limiting the data for respondents who answered the survey question without any help from others [see Additional file 2: Sensitivity analyses].

## Discussion

Using four waves of longitudinal data from the CLHLS sample of older Chinese adults, this study found that living arrangements were significantly associated with PWB. Older adults living with family or in an institution had better PWB than their counterparts living alone. In addition, we provided new evidence that these relationships were moderated by income sources after adjusting for socio-demographic factors, health behaviours and health status.

Our findings were consistent with other research [11, 36–38]. Both today and in the past, living with family is important because of the support that family provides and the influence of this support on the well-being of older individuals. Living with family usually refers to residing with a spouse, children or both. A spouse was considered to contribute more to the emotional well-being of their partner. Adult children were found to play a more important role in improving the well-being of a parent whose spouse has passed away [39]. Adult children were responsible for taking care of their parents [40] and assumed a strong role in providing their parents instrumental, emotional and financial support in the traditional Confucian culture [41]. By living with family, adult parents obtain not only daily care but also emotional nurturing from their families. Indeed, grandchildren have been an important part of the traditional family in China, especially in rural areas. With the progress of labour migration, left-behind children were naturally being cared for by their grandparents, which may also provide emotional comfort for older adults. Therefore, despite population migration and social transformation, living with family was still a popular pattern and was beneficial for the PWB of older Chinese adults.

In contrast with developed countries [15], residing in an institution was better than living alone for older Chinese adults. Institutionalized older adults, especially those who are very sick and disadvantaged, preferred to reside in the institution, as the availability of institutionalized care and facilities, which were evaluated positively by older adults, promoted their PWB. For those with limited or no family support, institutionalization may be beneficial to mitigate feelings of loneliness from social isolation and social shame [24].

We found that living alone was harmful to older adults’ PWB, which was inconsistent with other studies [21, 42, 43]. Those living alone were younger and most of them had better health status than their counterparts in our study. Lack of financial support, emotional comfort and care services are the three main problems for these persons [44]. Since mental health is a determinant of life satisfaction for older adults [45], society should pay more attention to these people.

Our study showed that income sources were significantly associated with the PWB of older Chinese adults. The results indicated that older adults supported by children or the government had lower PWB scores than those who were financially self-sufficient. Furthermore, older adults living with family and supported by the government had significantly higher PWB scores. This finding suggests that financial support from the government can increase older adults’ PWB resulting from living with family. Government support through Dibao could meet the basic needs of older adults by establishing a security net and mitigating the tensions among household members due to poverty [28]. In addition, being supported by their children was worse than supporting themselves for those living in an institution. In a culture emphasizing filial piety, adult children might be regarded as unfilial if they send their parents to an institution. However, most of those who were living in an institution and receiving support from the government were the “three-no” older adults, defined as having little or no income, no living children or relatives, and no physical ability to work [15], Because of shifts in family size, pension structure and funding, the demand for such institutions will gradually increase, and the government should increase the number of public institutions to supply more beds and promote private pensions to provide more choices for older adults.

Several limitations of this study should be noted. First, a potential bias may arise from sample attrition. Respondents were lost to follow-up when they were male, had a higher education level, and lived in an urban area. Most of those characteristics are positively associated with PWB. The results of sensitivity analysis showed that the estimated relationship between living arrangements and PWB remained nonsignificant, with adjustment for the dummy variable indicating death or loss to follow-up. Second, although random effects were added to the ordinal probit model, the self-selection problem regarding living arrangements for different PWB was not completely addressed. Third, although we used longitudinal data from the CLHLS, we should be cautious about causal inferences. Additional studies are warranted to examine the mechanisms of why living arrangements can affect PWB. Fourth, although we adjusted for as many available covariates as possible, the data limitations restricted us from including some potential confounders, such as wealth or family relationships, which may be associated with PWB.

## Conclusions

Despite these limitations, our analysis provides a significant contribution to the existing literature on the relationship between living arrangements and PWB in China. Understanding how living arrangements may affect PWB is critical in China, a society with the largest older population in the world. We recognize that living with family or in an institution leads to better PWB than does living alone. In addition, financial support from the government can moderate this association. Our study encourages future research to investigate the causal mechanisms through which living arrangements affect PWB.

## Supplementary information

**Additional file 1: Table S1.** Coefficients from random-effect ordinal probit models for psychological well-being stratified by income sources.

**Additional file 2: Table S2.** Coefficients from random-effect ordinal probit models for psychological well-being adjusting for sample attrition. **Table S3.** Coefficients from random-effect ordinal probit models for psychological well-being excluding the participants with full proxy responses.

## Data Availability

The CLHLS dataset is publicly available. Information about the data source and available data are found at https://www.icpsr.umich.edu/icpsrweb/DSDR/studies/36179. Researchers can obtain these data after submitting a data use agreement to the CLHLS team.

## Abbreviations

ADL: Activities of daily living
CLHLS: Chinese Longitudinal Healthy Longevity Survey
MMSE: Mini-Mental State Examination
PWB: Psychological well-being
