# Supplementary material for: A longitudinal analysis of the association between the living arrangements and psychological well-being of older Chinese adults: the role of income sources
Source: BMC Geriatr. 2019 Dec 10;19:347. doi: 10.1186/s12877-019-1371-0 (PMC6904999; doi:10.1186/s12877-019-1371-0)
Supplement: Supplementary file 1 — Additional file 1: Table S1. Coefficients from random-effect ordinal probit models for psychological well-being stratified by income sources. [file 12877_2019_1371_MOESM1_ESM.docx]

**Table S1: Coefficients from random-effect ordinal probit models for psychological well-being stratified by income sources**

| **Variables** | **Psychological well-being** | | |  |
| --- | --- | --- | --- | --- |
|  | **Financial self-support (n=9,190)** | **Children support**  **(n=19,508)** | **Government support**  **(n=2,201)** | |
| **Socio-demographic factors** | |  |  | |
| Age | 0.00 (0.00 - 0.01) ^*^ | 0.01 (0.01 - 0.01) ^***^ | 0.01 (0.01 - 0.02) ^***^ | |
| Wave | -0.01 (-0.01 - 0.00) | -0.00 (-0.01 - 0.00) | 0.01 (-0.00 - 0.03) | |
| Rural (vs. urban) | -0.20 (-0.26 - -0.15) ^***^ | -0.10 (-0.13 - -0.07) ^***^ | -0.08 (-0.18 - 0.01) | |
| Female (vs. male) | -0.12 (-0.18 - -0.07) ^***^ | -0.06 (-0.10 - -0.02) ^**^ | -0.06 (-0.17 - 0.06) | |
| Minority (vs. Han) | -0.03 (-0.16 - 0.09) | 0.03 (-0.03 - 0.10) | -0.20 (-0.42 - 0.02) | |
| Married (vs. unmarried) | 0.16 (0.10 - 0.22) ^***^ | 0.11 (0.06 - 0.15) ^***^ | 0.12 (-0.03 - 0.26) | |
| Child alive (vs. no child alive) | 0.05 (-0.06 - 0.15) | 0.04 (-0.02 - 0.11) | 0.11 (-0.01 - 0.23) | |
| Sibling alive (vs. no sibling alive) | 0.05 (-0.00 - 0.10) | 0.04 (0.01 - 0.08) ^*^ | 0.06 (-0.05 - 0.17) | |
| Education | 0.01 (0.01 - 0.01) ^***^ | 0.00 (-0.00 - 0.00) | 0.01 (-0.00 - 0.01) | |
| Professional occupation (vs. non-professional) | 0.17 (0.10 - 0.24) ^***^ | 0.10 (0.03 - 0.17) ^**^ | 0.06 (-0.08 - 0.20) | |
| Financially sufficient (vs. insufficient) | 0.66 (0.60 - 0.73) ^***^ | 0.45 (0.42 - 0.49) ^***^ | 0.42 (0.32 - 0.52) ^***^ | |
| Social support (vs. no) | 0.25 (0.15 - 0.36) ^***^ | 0.02 (-0.06 - 0.09) | 0.35 (0.20 - 0.50) ^***^ | |
| **Health behaviours** |  |  |  | |
| Current smoker (vs. no) | -0.03 (-0.09 - 0.03) | 0.09 (0.04 - 0.14) ^***^ | 0.09 (-0.04 - 0.22) | |
| Current drinker (vs. no) | 0.16 (0.11 - 0.22) ^***^ | 0.07 (0.02 - 0.11) ^**^ | 0.16 (0.04 - 0.28) ^*^ | |
| Regular exercise (vs. no) | 0.34 (0.29 - 0.39) ^***^ | 0.26 (0.22 - 0.30) ^***^ | 0.30 (0.19 - 0.40) ^***^ | |
| **Health status** |  |  |  | |
| Chronic diseases | -0.09 (-0.12 - -0.06) ^***^ | -0.08 (-0.10 - -0.06) ^***^ | -0.11 (-0.17 - -0.06) ^***^ | |
| ADL disability | -0.04 (-0.08 - 0.01) | 0.02 (-0.01 - 0.04) | 0.06 (-0.01 - 0.14) | |
| Cognitively impaired (vs. non-impaired) | 0.74 (0.65 - 0.83) ^***^ | 0.46 (0.42 - 0.50) ^***^ | 0.37 (0.26 - 0.49) ^***^ | |
| **Living arrangements (vs. living alone)** | | |  |  |
| With family | 0.26 (0.18 - 0.34) ^***^ | 0.31 (0.26 - 0.36) ^***^ | 0.33 (0.20 - 0.47) ^***^ | |
| Institution | 0.27 (0.12 - 0.43) ^***^ | 0.16 (0.03 - 0.29) ^*^ | 0.42 (0.27 - 0.57) ^***^ | |
| Variance of random effect | 0.18 (0.15 - 0.23)^***^ | 0.15 (0.12 - 0.19)^***^ | 0.20 (0.11 - 0.38) | |
| LR test | 112.69^***^ | 135.91^***^ | 15.06^***^ | |

*Notes.* ADL=activities of daily living; LR=likelihood ratio; ^*^*P*< .05, ^**^*P*< .01, ^***^*P*< .001.
