# Supplementary material for: A longitudinal analysis of the association between the living arrangements and psychological well-being of older Chinese adults: the role of income sources
Source: BMC Geriatr. 2019 Dec 10;19:347. doi: 10.1186/s12877-019-1371-0 (PMC6904999; doi:10.1186/s12877-019-1371-0)
Supplement: Supplementary file 2 — Additional file 2: Table S2. Coefficients from random-effect ordinal probit models for psychological well-being adjusting for sample attrition. Table S3. Coefficients from random-effect ordinal probit models for psychological well-being excluding the participants with full proxy responses. [file 12877_2019_1371_MOESM2_ESM.docx]

**Sensitivity analyses**

**Table S2: Coefficients from random-effect ordinal probit models for psychological well-being adjusting for sample attrition**

| **Variables** | **Psychological well-being** | | |
| --- | --- | --- | --- |
|  | **Model 1** | **Model 2** | **Model 3** |
| **Socio-demographic factors** |  |  |  |
| Age | 0.01 (0.01 - 0.01) ^***^ | 0.01 (0.01 - 0.01) ^***^ | 0.01 (0.01 - 0.01) ^***^ |
| Wave | -0.01 (-0.01 - -0.00) ^**^ | -0.01 (-0.01 - -0.00) ^*^ | -0.01 (-0.01 - -0.00) ^*^ |
| Rural (vs. urban) | -0.15 (-0.18 - -0.12)^***^ | -0.13 (-0.15 - -0.10) ^***^ | -0.13 (-0.16 - -0.10) ^***^ |
| Female (vs. male) | -0.10 (-0.13 - -0.07)^***^ | -0.08 (-0.12 - -0.05) ^***^ | -0.08 (-0.11 - -0.05) ^***^ |
| Minority (vs. Han) | 0.00 (-0.05 - 0.06) | 0.01 (-0.05 - 0.06) | 0.01 (-0.05 - 0.06) |
| Married (vs. unmarried) | 0.14 (0.11 - 0.18) ^***^ | 0.12 (0.08 - 0.15) ^***^ | 0.12 (0.08 - 0.15) ^***^ |
| Child alive (vs. no child alive) | 0.08 (0.03 - 0.13) ^**^ | 0.07 (0.02 - 0.12) ^**^ | 0.08 (0.03 - 0.12) ^**^ |
| Sibling alive (vs. no sibling alive) | 0.05 (0.02 - 0.07) ^**^ | 0.04 (0.02 - 0.07) ^**^ | 0.04 (0.02 - 0.07) ^**^ |
| Education | 0.00 (0.00 - 0.01) ^***^ | 0.00 (0.00 - 0.01) ^***^ | 0.00 (0.00 - 0.01) ^***^ |
| Professional occupation (vs. non-professional) | 0.15 (0.10 - 0.19) ^***^ | 0.13 (0.09 - 0.18) ^***^ | 0.13 (0.09 - 0.18) ^***^ |
| Financially sufficient (vs. insufficient) | 0.50 (0.47 - 0.53) ^***^ | 0.49 (0.46 - 0.52) ^***^ | 0.49 (0.46 - 0.52) ^***^ |
| Social support (vs. no) | 0.11 (0.06 - 0.17) ^***^ | 0.12 (0.06 - 0.17) ^***^ | 0.11 (0.06 - 0.17) ^***^ |
| **Health behaviours** |  |  |  |
| Current smoker (vs. no) | 0.04 (0.00 - 0.07) ^*^ | 0.04 (0.01 - 0.08) ^*^ | 0.04 (0.01 - 0.08) ^*^ |
| Current drinker (vs. no) | 0.10 (0.06 - 0.13) ^***^ | 0.09 (0.06 - 0.13) ^***^ | 0.09 (0.06 - 0.13) ^***^ |
| Regular exercise (vs. no) | 0.29 (0.26 - 0.32) ^***^ | 0.28 (0.26 - 0.31) ^***^ | 0.29 (0.26 - 0.31) ^***^ |
| **Health status** |  |  |  |
| Chronic diseases | -0.08 (-0.10 - -0.06)^***^ | -0.08 (-0.10 - -0.07) ^***^ | -0.08 (-0.10 - -0.07) ^***^ |
| ADL disability | 0.02 (0.00 - 0.04) ^*^ | 0.02 (0.00 - 0.04) ^*^ | 0.02 (0.00 - 0.04) ^*^ |
| Cognitively impaired (vs. non-impaired) | 0.51 (0.47 - 0.54) ^***^ | 0.51 (0.48 - 0.55) ^***^ | 0.51 (0.48 - 0.55) ^***^ |
| **Living arrangements (vs. living alone)** | | |  |
| With family | 0.31 (0.27 - 0.35) ^***^ | 0.31 (0.28 - 0.35) ^***^ | 0.29 (0.24 - 0.33) ^***^ |
| Institution | 0.27 (0.19 - 0.34) ^***^ | 0.29 (0.21 - 0.36) ^***^ | 0.34 (0.19 - 0.49) ^***^ |
| **Income sources (vs. financial self-support)** | | | |
| Children support |  | -0.14 (-0.20 - -0.08) ^***^ | -0.23 (-0.35 - -0.12) ^***^ |
| Government support |  | -0.12 (-0.15 - -0.09) ^***^ | -0.08 (-0.16 - -0.00) ^*^ |
| **Living arrangements^*^Income sources** | | | |
| With family^*^children support |  |  | 0.04 (-0.04 - 0.13) |
| With family^*^government support |  |  | 0.16 (0.03 - 0.28) ^*^ |
| Institution^*^ children support |  |  | -0.21 (-0.41 - -0.02) ^*^ |
| Institution^*^ government support |  |  | 0.12 (-0.08 - 0.31) |
| Censored at next wave (vs.no) | -0.07 (-0.10 - -0.04) ^***^ | -0.07 (-0.10 - -0.04)^***^ | -0.07 (-0.10 - -0.04)^***^ |
| Variance of random effect | 0.18 (0.16 - 0.20)^***^ | 0.17 (0.15 - 0.20)^***^ | 0.17 (0.15 - 0.20)^***^ |
| LR test | 355.34^***^ | 343.82^***^ | 343.41^***^ |

*Notes.* ADL=activities of daily living; LR=likelihood ratio; ^*^*P*< .05, ^**^*P*< .01, ^***^*P*< .001.

**Table S3. Coefficients from random-effect ordinal probit models for psychological well-being excluding the participants with full proxy responses**

| **Variables** | **Psychological well-being** | | |
| --- | --- | --- | --- |
|  | **Model 1** | **Model 2** | **Model 3** |
| **Socio-demographic factors** |  |  |  |
| Age | 0.01 (0.01 - 0.01) ^***^ | 0.01 (0.01 - 0.01) ^***^ | 0.01 (0.01 - 0.01) ^***^ |
| Wave | -0.00 (-0.01 - -0.00) ^*^ | -0.00 (-0.01 - 0.00) | -0.00 (-0.01 - 0.00) |
| Rural (vs. urban) | -0.15 (-0.18 - -0.12) ^***^ | -0.13 (-0.15 - -0.10)^***^ | -0.13 (-0.16 - -0.10) ^***^ |
| Female (vs. male) | -0.10 (-0.13 - -0.07) ^***^ | -0.08 (-0.11 - -0.05)^***^ | -0.08 (-0.11 - -0.05) ^***^ |
| Minority (vs. Han) | 0.01 (-0.05 - 0.06) | 0.01 (-0.05 - 0.07) | 0.01 (-0.05 - 0.07) |
| Married (vs. unmarried) | 0.14 (0.11 - 0.18) ^***^ | 0.12 (0.08 - 0.15) ^***^ | 0.12 (0.08 - 0.15) ^***^ |
| Child alive (vs. no child alive) | 0.08 (0.03 - 0.13) ^**^ | 0.07 (0.02 - 0.12) ^**^ | 0.08 (0.03 - 0.13) ^**^ |
| Sibling alive (vs. no sibling alive) | 0.05 (0.02 - 0.08) ^***^ | 0.05 (0.02 - 0.08) ^**^ | 0.05 (0.02 - 0.08) ^**^ |
| Education | 0.00 (0.00 - 0.01) ^***^ | 0.00 (0.00 - 0.01) ^***^ | 0.00 (0.00 - 0.01) ^***^ |
| Professional occupation (vs. non-professional) | 0.15 (0.10 - 0.19) ^***^ | 0.13 (0.09 - 0.18) ^***^ | 0.13 (0.09 - 0.18) ^***^ |
| Financially sufficient (vs. insufficient) | 0.50 (0.46 - 0.53) ^***^ | 0.49 (0.46 - 0.52) ^***^ | 0.49 (0.46 - 0.52) ^***^ |
| Social support (vs. no) | 0.11 (0.06 - 0.17) ^***^ | 0.12 (0.06 - 0.17) ^***^ | 0.11 (0.06 - 0.17) ^***^ |
| **Health behaviours** |  |  |  |
| Current smoker (vs. no) | 0.04 (0.00 - 0.07) ^*^ | 0.04 (0.01 - 0.08) ^*^ | 0.04 (0.01 - 0.08) ^*^ |
| Current drinker (vs. no) | 0.10 (0.06 - 0.13) ^***^ | 0.10 (0.06 - 0.13) ^***^ | 0.10 (0.06 - 0.13) ^***^ |
| Regular exercise (vs. no) | 0.30 (0.27 - 0.32) ^***^ | 0.29 (0.26 - 0.32) ^***^ | 0.29 (0.26 - 0.32) ^***^ |
| **Health status** |  |  |  |
| Chronic diseases | -0.08 (-0.10 - -0.07) ^***^ | -0.08 (-0.10 - -0.07)^***^ | -0.08 (-0.10 - -0.07) ^***^ |
| ADL disability | 0.02 (-0.00 - 0.04) | 0.02 (-0.00 - 0.04) | 0.02 (-0.00 - 0.04) |
| Cognitively impaired (vs. non-impaired) | 0.50 (0.47 - 0.54) ^***^ | 0.51 (0.47 - 0.54) ^***^ | 0.51 (0.47 - 0.54) ^***^ |
| **Living arrangements (vs. living alone)** | | |  |
| With family | 0.31 (0.27 - 0.35) ^***^ | 0.31 (0.27 - 0.35) ^***^ | 0.28 (0.24 - 0.33) ^***^ |
| Institution | 0.26 (0.19 - 0.34) ^***^ | 0.28 (0.20 - 0.36) ^***^ | 0.34 (0.19 - 0.49) ^***^ |
| **Income sources (vs. financial self-support)** | | | |
| Children support |  | -0.12 (-0.16 - -0.09)^***^ | -0.08 (-0.15 - -0.00) ^*^ |
| Government support |  | -0.14 (-0.20 - -0.08)^***^ | -0.23 (-0.35 - -0.12) ^***^ |
| **Living arrangements^*^Income sources** | | | |
| With family^*^children support |  |  | 0.05 (-0.03 - 0.13) |
| With family^*^government support |  |  | 0.16 (0.03 - 0.28) ^*^ |
| Institution^*^ children support |  |  | -0.22 (-0.41 - -0.02) ^*^ |
| Institution^*^ government support |  |  | 0.12 (-0.08 - 0.32) |
| Variance of random effect | 0.18 (0.16 - 0.20)^***^ | 0.17 (0.15 - 0.20)^***^ | 0.17 (0.15 - 0.20)^***^ |
| LR test | 355.33^***^ | 343.54^***^ | 343.01^***^ |

*Notes.* ADL=activities of daily living; LR=likelihood ratio; ^*^*P*< .05, ^**^*P*< .01, ^***^*P*< .001.
